# Bright GFP with subnanosecond fluorescence lifetime

**DOI:** 10.1038/s41598-018-31687-w

**Published:** 2018-09-05

**Authors:** Anastasia V. Mamontova, Ilya D. Solovyev, Alexander P. Savitsky, Alexander М. Shakhov, Konstantin A. Lukyanov, Alexey M. Bogdanov

**Affiliations:** 1 Shemyakin-Ovchinnikov Institute of Bioorganic Chemistry, Moscow, Russia; 2 Bach Institute of Biochemistry, Research Center of Biotechnology of the Russian Academy of Sciences, Moscow, Russia; 3 Department of Chemistry, Lomonosov Moscow State University, Moscow, Russia; 4 Semenov Institute of Chemical Physics, Moscow, Russia; 5 Moscow Institute of Physics and Technology, Dolgoprudny, Moscow region Russia

## Abstract

Fluorescence lifetime imaging microscopy (FLIM) measures fluorescence decay rate at every pixel of an image. FLIM can separate probes of the same color but different fluorescence lifetimes (FL), thus it is a promising approach for multiparameter imaging. However, available GFP-like fluorescent proteins (FP) possess a narrow range of FLs (commonly, 2.3–3.5 ns) which limits their applicability for multiparameter FLIM. Here we report a new FP probe showing both subnanosecond fluorescence lifetime and exceptional fluorescence brightness (80% of EGFP). To design this probe we applied semi-rational amino acid substitutions selection. Critical positions (Thr65, Tyr145, Phe165) were altered based on previously reported effect on FL or excited state electron transfer. The resulting EGFP triple mutant, BrUSLEE (Bright Ultimately Short Lifetime Enhanced Emitter), allows for both reliable detection of the probe and recording FL signal clearly distinguishable from that of the spectrally similar commonly used GFPs. We demonstrated high performance of this probe in multiparameter FLIM experiment. We suggest that amino acid substitutions described here lead to a significant shift in radiative and non-radiative excited state processes equilibrium.

## Introduction

Since the cloning of the avGFP gene in the early 1990s, fluorescent proteins (FP) have become an indispensable instrument in biology^1^. FP-based multiparameter live cell imaging is of enormous importance in deciphering complex biological phenomena. There are several key characteristics determining FP practical utility^2^. Among them color holds a specific place being a critical factor in such qualitative imaging approaches as the multicolor labeling. To date, only 3–5 colors within a wide collection of FP variants can be reliably distinguished while visualized simultaneously. Fluorescence lifetime imaging microscopy (FLIM) allows unmixing of signals generated by the probes of the same color^3,4^. Thus, a diversity of the independently detectable markers could potentially be greatly extended.

Fluorescence lifetimes (FL) of most FPs fall within range from 2.3 to 3.5 ns, although extreme values from 0.7 to over 5.0 ns were documented^5^. A significant progress has been achieved in development of FPs with long fluorescence lifetimes, e.g., cyan mTurquoise2 (4.0 ns)^6^, green WasCFP and NowGFP (5.1 ns)^5,7^, red mScarlet (3.9 ns)^8^. At the same time, a field of FPs with subnanosecond lifetimes remains almost unexplored. The main reason is that fluorescence lifetime shortening is normally correlated with a proportional decrease of fluorescence quantum yield (http://www.fpvis.org/FP.html). Thus, FPs with a short FL (<1.0 ns) possess very low quantum yield (<0.1). In particular, mGarnet2 and TagRFP675 – FPs with the shortest fluorescence lifetimes reported to date (760 and 900 ps, respectively) and red emission – have quantum yield of less than 10%^9,10^. Also, to the best of our knowledge, FPs with subnanosecond fluorescence lifetimes in other parts of spectrum have not been previously described. Particularly, green FPs with subnanosecond lifetimes reported to date are represented by the extremely dim mutants or even chromoproteins^11^. The low fluorescence brightness of such probes complicates their application in multiparameter FLIM.

## Results and Discussion

**Figure 1:**
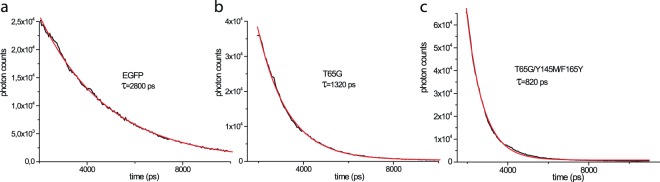
Fluorescence decay curves of the purified EGFP (**a**) and its mutants (**b**,**c**) recorded using two-photon excitation in aqueous solution, and their single-exponential fits. Experimental decay curves are shown in black, exponential fits – in red. Lifetimes (τ) are shown under the protein names.

Here we applied semi-rational molecular evolution to generate a bright EGFP variant with subnanosecond lifetime. We started with the evaluation of EGFP-T65G mutant as this substitution was shown to decrease FL in a related GFP variant^12^. Indeed, EGFP-T65G possessed shorter lifetime and lower quantum yield (QY) compared to the parental EGFP (1.3 ns vs 2.8 ns in EGFP, Table 1 and Fig. 1a,b).

**Table 1:** Spectral properties and fluorescence lifetimes of EGFP and its mutants.

Fluorescent protein	λex/λem, nm	EC, M^−1^ cm^−1^	QY^a^	Relative brightness, %^b^	Fluorescence lifetime, ps	Relative photostability, %^c^	
						*in vitro*	*in cellulo*
EGFP	489/509	55000	0.60	100	2800 ± 70	100 ± 8	100 ± 20
EGFP-T65G	488/508	70000 ± 1500	0.06 ± 0.01	13 ± 2	1320 ± 30	180 ± 25	240 ± 63
EGFP-T65G/Y145M	484/508	84500 ± 1400	0.08 ± 0.01	20 ± 3	820 ± 8	175 ± 12	700 ± 280
EGFP-T65G/Y145M/F165Y (BrUSLEE)	487/509	86000 ± 1200	0.30 ± 0.04	78 ± 12	820 ± 5	190 ± 4	230 ± 45

Standard deviations (N = 3 for EC and QY, n = 3 for *in vitro*, n = 10 for *in cellulo* photostability) are shown. Exponential approximation errors are shown as the experimental uncertainties for fluorescence lifetime.

^a^For EGFP absolute quantum yield is shown, for the mutants quantum yields measured relative to the equally absorbing EGFP (see Materials and Methods) are shown.

^b^Relative brightness is calculated as a product of molar extinction coefficient and fluorescence quantum yield and given compared to the brightness of EGFP.

^c^Relative photostability is the half-bleaching time of the FP of interest relative to that of EGFP illuminated under the same conditions. Left column corresponds *in vitro* photostability of the purified protein in PBS, right one – *in cellulo* photostability measured in HEK293 cells expressing the FP of interest.

Next we applied saturation mutagenesis at positions 145 and 165. These positions were previously found to be important for excited state electron transfer (ESET)^13^. In turn, ESET can have a great impact on FL^4^. Several obtained T65G/Y145X mutants demonstrated FL of approximately 800 ps (partially shown in Table 1), revealing a role of the 145^th^ position as a gateway to FL reduction. However, their QYs were an order of magnitude lower than that of EGFP. Finally, a triple mutant T65G/Y145M/F165Y called BrUSLEE (Bright Ultimately Short Lifetime Enhanced Emitter) possessed both high brightness (~80% of EGFP, QY = 0.3, EC = 86000 M^−1^ cm^−1^) and short FL (~800 ps, Fig. 1c and Table 1). Being spectrally similar to the parental EGFP (Supplementary Fig. 1), both T65G and BrUSLEE demonstrated enhanced photostability in comparison to EGFP (Table 1). It is worthy of note that we detected circa twofold reduced photobleaching rates not only for the purified proteins but for the proteins expressed in live mammalian cells as well.

**Figure 2:**
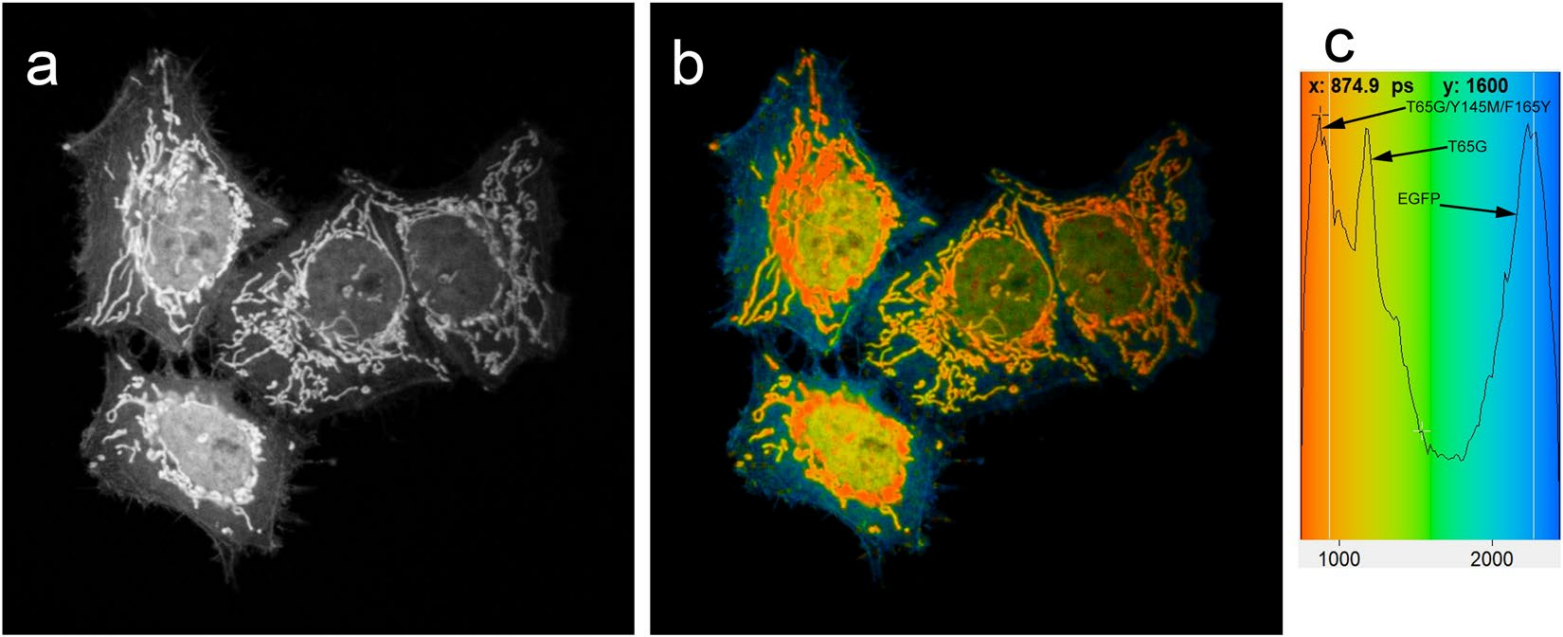
Fluorescence and fluorescence lifetime imaging microscopy of live HeLa cells expressing EGFP-actin (mainly in cytoplasm; Tm ~2.2 ns), EGFP-T65G-histone 2B (in nucleus; Tm ~1.1 ns), BrUSLEE-mito (in mitochondria; Tm ~0.8 ns). (**a**) Fluorescence intensity image in grayscale, (**b**) Color-coded combined intensity + lifetime image, brightness represents photon counts, color represents fluorescence lifetime, (**c**) Color legend for the fluorescence lifetime image with a histogram of lifetime distribution (legend range is 780–2350 ps). Single-photon fluorescence excitation at 488 nm was used to acquire these images. Fluorescence decay data and analysis are shown in Supplementary Fig. 2.

To test applicability of the new mutants for multiparameter FLIM in green channel, EGFP-actin, T65G-histone H2B, and BrUSLEE-mito were co-expressed and visualized simultaneously in live mammalian cells. These three FPs possess extremely similar fluorescence spectra (Supplementary Fig. 1) and cannot be discriminated by common fluorescence microscopy. However, they were clearly distinguishable with FLIM (Fig. 2 and Supplementary Figs 2 and 3). We detected considerable difference in FL values *in cellulo* compared to that of the purified proteins (2.2 vs 2.8 ns for EGFP, 0.85 vs 1.3 ns for T65G and 0.6 vs 0.8 ns for BrUSLEE, Supplementary Fig. 2). Such FL shortening in cellular environment has been described for EGFP and some other FPs^5,14,15^. Also shifts in experimental lifetime values of the same fluorophore may be attributed to the different hardware sets used.

To adequately interpret particular roles of the introduced amino acid substitutions and to draw a complete mechanistic model explaining observed relationships between fluorescence lifetime and quantum yield in the mutant described, both sophisticated additional experiments and theoretical computations are required. However, we hypothesize that the mutations substantially altered excited-state non-radiative processes (such as electron transfer and/or conformation changes) and radiative decay efficiencies. Taking into account equations (see Eqs 1 and 2) connecting quantum yield and experimental lifetime through the radiative and non-radiative rate constants, one can do a first approximation of these rates in EGFP and its triple mutant. Thus, radiative rate in EGFP is circa two times lower, whereas non-radiative rate – 6 times lower than corresponding rates in BrUSLEE.

1 $$\Phi =\frac{{k}_{r}}{{k}_{r}+{k}_{nr}}$$

2 $$\tau =\frac{1}{{k}_{r}+{k}_{nr}}$$

Relationships between (1) a fluorescence quantum yield (Ф) and (2) a fluorescence lifetime (τ) and rate constants of the radiative (k_r_) and non-radiative (k_nr_) processes in a fluorophore excited state.

To conclude, here we reported two EGFP mutations for FL shortening (T65G and Y145M), and the third F165Y mutation restoring protein brightness. The resulting mutant EGFP-T65G/Y145M/F165Y aka BrUSLEE possesses a unique combination of short FL and high QY, with fluorescence brightness comparable to that of the commonly used EGFP. This FP appears to be a probe of choice for multiparameter FLIM together with regular green FPs with long FL. Also, the short FL of BrUSLEE makes it possible to use high repetition rate (>80 MHz) excitation sources for faster imaging^16^. Our finding breaks the stereotype of inescapable connection between short FL and low QY in fluorescent protein probes and calls for development of a panel of bright subnanosecond FPs of different colors.

## Methods

### Spectroscopy and fluorescence brightness evaluation

For absorbance and fluorescence excitation-emission spectra measurements, Cary 100 UV/VIS spectrophotometer and Cary Eclipse fluorescence spectrophotometer (Varian) were used. Fluorescence brightness was evaluated as a product of molar extinction coefficient by quantum yield multiplication. Measurements on all native proteins were carried out in phosphate buffered saline (PBS, pH 7.4, GIBCO). For molar extinction coefficient determination, we relied on measuring mature chromophore concentration. EGFP and its mutants were alkali-denatured in 1 M NaOH. Under these conditions GFP-like chromophore is known to absorb at 447 nm with extinction coefficient of 44,000 M^−1^ cm^−1^. Based on the absorption of the native and alkali-denatured proteins, molar extinction coefficients for the native states were calculated. For determination of the quantum yield, the areas under fluorescence emission spectra of the mutants were compared with equally absorbing EGFP (quantum yield 0.60).

### Fluorescence lifetime imaging microscopy of the purified proteins upon two-photon excitation

Femtosecond laser pulses (80 MHz repetition rate, 100 fs, up to 25 nJ per pulse) were generated by a Ti:Sapphire oscillator (Tsunami, Spectra-Physics) pumped by a green Nd:YVO_4_ CW laser (532 nm, Millennia Prime 6sJ, Spectra-Physics). Femtosecond laser beam was coupled to an inverted optical microscope Olympus IX71 by Thorlabs FESH0750 dielectric filter mounted at 45° and then focused by objective lens (40 × 0.75NA UPlanFLN, Olympus) on a sample, which was placed on a 3-axis stage. The samples were prepared as droplets of the purified fluorescent proteins dissolved in phosphate buffered saline (PBS, pH 7.4, GIBCO) applied onto a standard 24 × 24 mm cover glass (Heinz Herenz, Germany).

The average laser power reaching the sample was precisely tuned with a polarizing attenuator consisting of a half-wave plate and a polarizing cube installed in front of microscope coupling dielectric filter. Two-photon excitation was typically performed at 900 nm and average laser power of 10–20 mW. The SF10 prism compressor was used to compensate for the group velocity dispersion in the objective lens and other optical elements.

Fluorescence was excited by two-photon absorption of femtosecond laser, passed back through the objective lens and laser coupling filter and then was directed to the input of Acton SP300i monochromator with two separate outputs. PI-MAX 2 CCD camera (Princeton Instruments) at the first output was employed for the fluorescence spectra registration. Photomultiplier tube of the time-correlated single photon counting system SPC-730 (Becker & Hickl GmbH) at the second output detected the fluorescence decay kinetics in the 510 nm–530 nm band. Fluorescence decay data were primarily acquired using SPCImage software (Becker & Hickl, Germany) and then exported in ASCII format and analyzed using Origin Pro 9 software (OriginLab, USA).

### Fluorescence lifetime imaging microscopy of live HeLa Kyoto cells with single-photon excitation

FLIM of live HeLa Kyoto cells was performed using Nikon TE-2000U microscope with Nikon 100x S Fluor 0.5–1.3 oil iris objective, equipped with the Becker&Hickl DCS-120 scanning confocal module and HPM-100-40 detector. For fluorescence excitation, Fianium WhiteLase SC-450-6 laser at a repetition rate of 60 MHz was used. Average input laser power was 1.5 mW, 488 nm laser line was generated by AOTF. To precisely adjust irradiation intensity continuously variable neutral density filters were used. Fluorescence emission signal was filtered by HQ495LP + HQ525/50 filter set (Chroma). Fluorescent images/lifetime data were acquired and analyzed using SPCImage software (Becker & Hickl, Germany).

### Protein expression and purification

EGFP and its mutants (EGFP-T65G and EGFP-T65G/Y145M/F165Y) were cloned into the pQE30 vector (Qiagen) with an N-terminal 6His tag, expressed in *E*. *coli* XL1 Blue strain (Invitrogen) and purified using TALON metal-affinity resin (Clontech). For mammalian cell expression, pTagRFP-mito, pmKate2-H2B vector backbones (Evrogen) and pEGFP-actin (Clontech) were used. EGFP-T65G was cloned into pmKate2-H2B in place of mKate2, EGFP T65G/Y145M/F165Y was cloned into pTagRFP-mito in place of TagRFP. HeLa Kyoto cells (ATCC) were co-transfected with the above listed constructs to obtain transient protein expression.

### Mammalian cell culture and transfection

HeLa Kyoto cell line (ATCC) was used. Cells were co-transfected with pEGFP-actin, pEGFP-T65G-H2B and pEGFP-T65G/Y145M/F165Y-mito using FuGene6 reagent (Promega) and grown in DMEM (Paneco) containing 10% FBS (Sigma). Same medium was used for imaging. Live cells were imaged 36 h post transfection using Leica AF6000 LX fluorescence microscope or Nikon TE-2000U-based FLIM microscope at room temperature.

### Site-directed mutagenesis

EGFP-T65G and EGFP-T65G/Y145M/F165Y mutants were generated using overlap-extension PCR technique with the following oligonucleotide set containing the appropriate substitutions: forward 5′-ATGCGGATCCATGGTGAGCAAGGGCGAG-3′, reverse 5′-ATGCAAGCTTTTACTTGTACAGCTCGTC-3′ and forward 5′-ACCACCCTGGGCTACGGCGTG-3′ and reverse 5′-CACGCCGTAGCCCAGGGTGGT-3′ for EGFP-T65G; forward 5′-ATGCGGATCCATGGTGAGCAAGGGCGAG-3′, reverse 5′-ATGCAAGCTTTTACTTGTACAGCTCGTC-3′, forward 5′-ACCACCCTGGGCTACGGCGTG-3′ and reverse 5′-CACGCCGTAGCCCAGGGTGGT-3′, forward 5′-GAGTACAACATGAACAGCCAC-3′ and reverse 5′-GTGGCTGTTTACGTTGTACTC-3′ and forward 5′-AAGGTGAACTACAAGATCCGC-3′ and reverse 5′-GCGGATCTTGTAGTTCACCTT-3′ for EGFP T65G/Y145M/F165Y. For bacterial expression, a PCR-amplified BamHI/HindIII fragment encoding respective mutant was cloned into the pQE30 vector (Qiagen). For mammalian expression, PCR-amplified (with 5′-CAGTACCGGTCGCCACCATGGTGAGCAAGGGCGAGGAGCTG-3′ and 5′-GATCGCGGCCGCTCACTTGTACAGCTCGTCCATGCCG-3′) AgeI/NotI fragments encoding T65G/Y145M/F165Y and T65G were cloned into pTagRFP-mito (Evrogen) and pmKate2-H2B (Evrogen) instead of the original FP genes, respectively.

## Electronic supplementary material

Supplementary Information

## Data Availability

The datasets generated during and/or analysed during the current study are available from the corresponding author on reasonable request.
