# Supplementary material for: Bright GFP with subnanosecond fluorescence lifetime
Source: Sci Rep. 2018 Sep 5;8:13224. doi: 10.1038/s41598-018-31687-w (PMC6125319; doi:10.1038/s41598-018-31687-w)
Supplement: Supplementary file 1 — Supplementary Information [file 41598_2018_31687_MOESM1_ESM.pdf]

Bright GFP with subnanosecond fluorescence lifetime

Anastasia V Mamontova, Ilya D Solovyev, Alexander P Savitsky, Alexander M Shakhov, Konstantin A Lukyanov & Alexey M Bogdanov

Supplementary figures and text

**Supplementary Figure 1**	Absorption and fluorescence spectra of EGFP and mutants.
**Supplementary Figure 2**	Fluorescence decay data and data analysis for live HeLa cells FLIM image.
**Supplementary Figure 3**	Color-coded FLIM images of live HeLa cells expressing EGFP-actin, EGFP T65G-histone 2B, EGFP-T65G/Y145M /F165Y-mito.
**Supplementary Methods**	

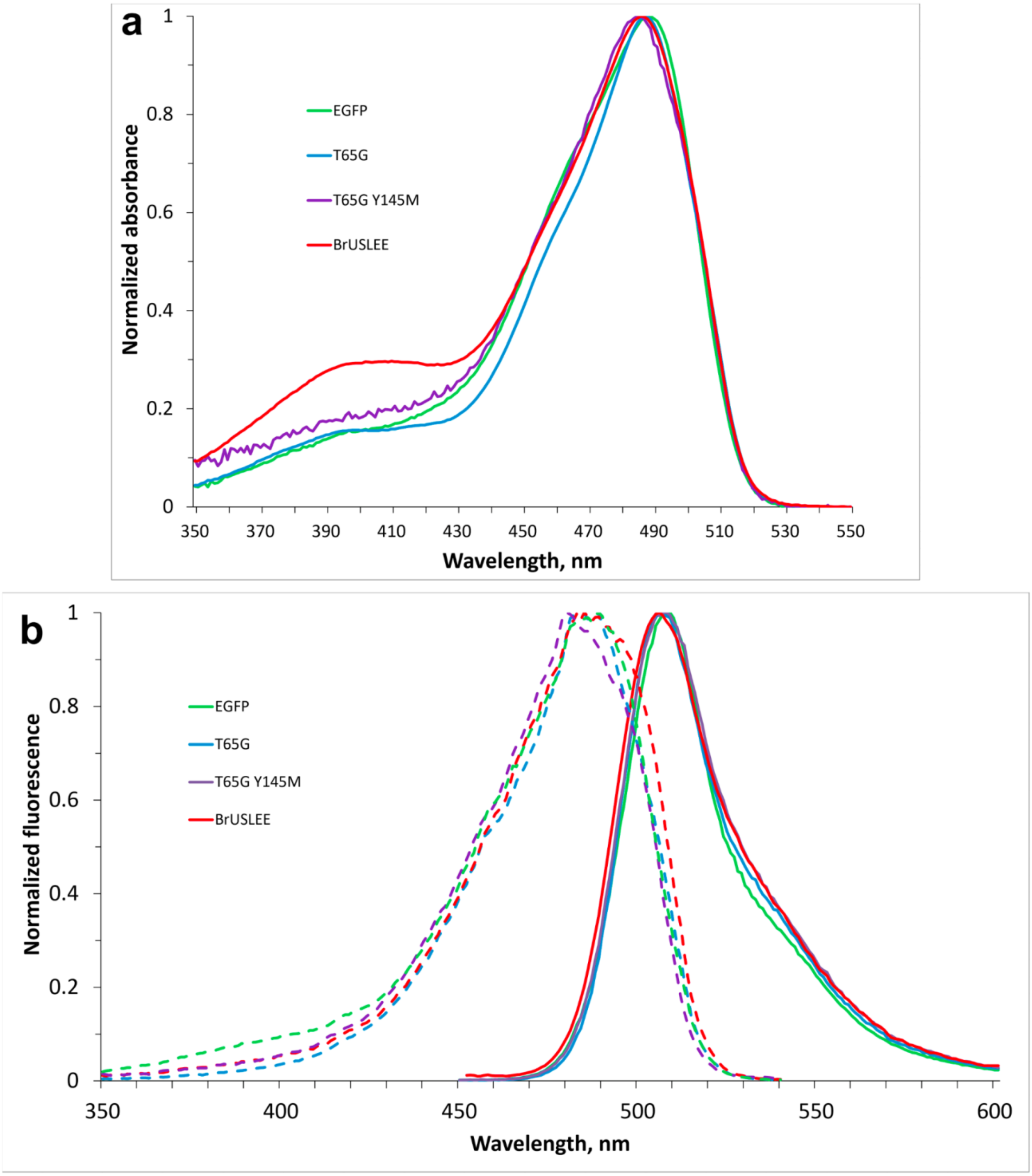

Supplementary Figure 1 | Absorption (a) and fluorescence (b) spectra of EGFP and mutants. In the fluorescence graph, dashed lines show fluorescence excitation, solid lines – fluorescence emission.

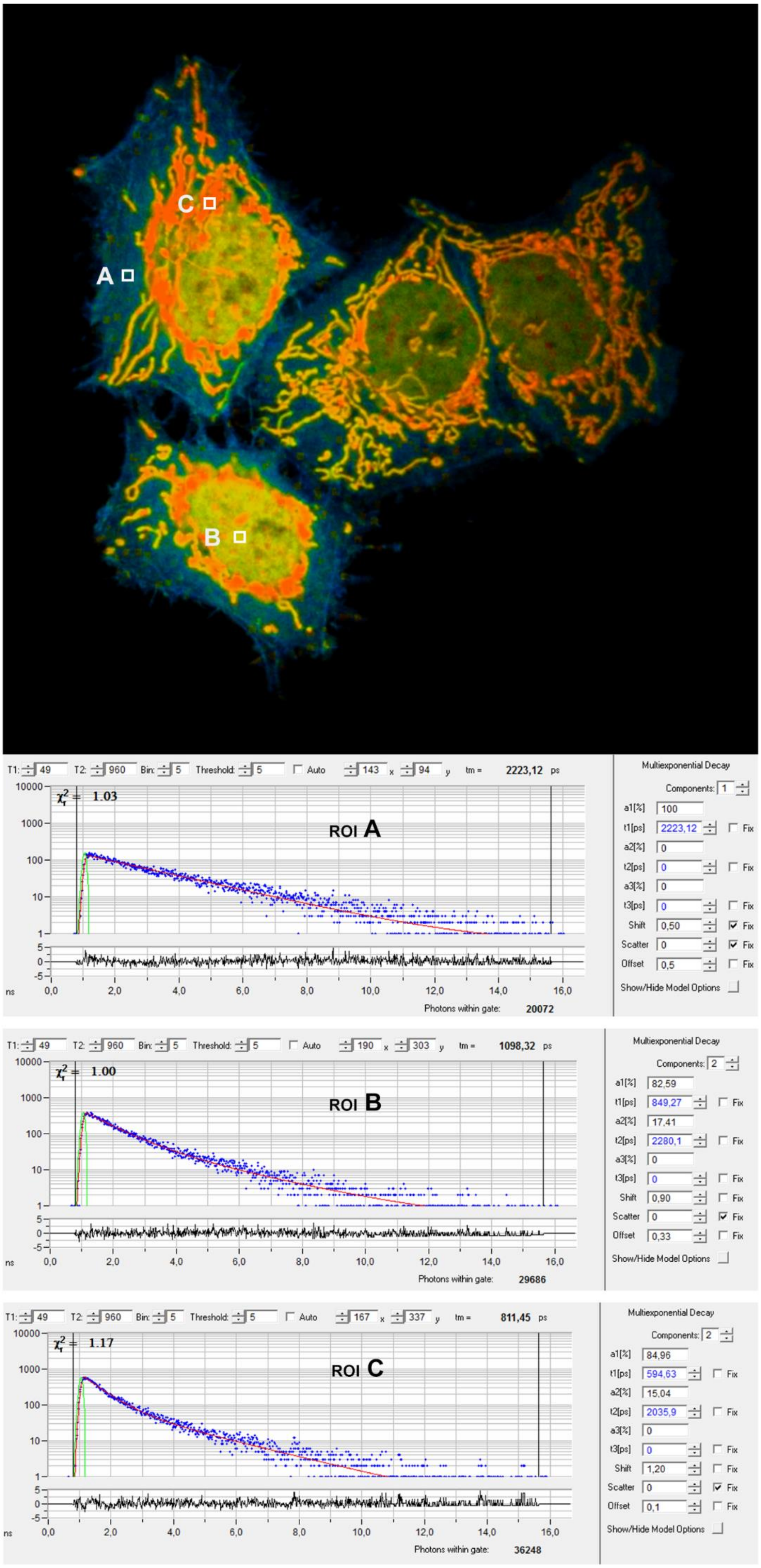
A
B
C
T1: 49 T2: 960 Bin: 5 Threshold: 5 Auto 143 x 94 y tm = 2223,12 ps
χ_r^2 = 1.03
ROI A
Photons within gate: 20072
Multiexponential Decay
Components: 1
a1[%] 100
t1[ps] 2223,12 Fix
a2[%] 0
t2[ps] 0 Fix
a3[%] 0
t3[ps] 0 Fix
Shift 0,50 Fix
Scatter 0 Fix
Offset 0,5 Fix
Show/Hide Model Options
T1: 49 T2: 960 Bin: 5 Threshold: 5 Auto 190 x 303 y tm = 1098,32 ps
χ_r^2 = 1.00
ROI B
Photons within gate: 29686
Multiexponential Decay
Components: 2
a1[%] 82,59
t1[ps] 849,27 Fix
a2[%] 17,41
t2[ps] 2280,1 Fix
a3[%] 0
t3[ps] 0 Fix
Shift 0,90 Fix
Scatter 0 Fix
Offset 0,33 Fix
Show/Hide Model Options
T1: 49 T2: 960 Bin: 5 Threshold: 5 Auto 167 x 337 y tm = 811,45 ps
χ_r^2 = 1.17
ROI C
Photons within gate: 36248
Multiexponential Decay
Components: 2
a1[%] 84,96
t1[ps] 594,63 Fix
a2[%] 15,04
t2[ps] 2035,9 Fix
a3[%] 0
t3[ps] 0 Fix
Shift 1,20 Fix
Scatter 0 Fix
Offset 0,1 Fix
Show/Hide Model Options
ns 0,0 2,0 4,0 6,0 8,0 10,0 12,0 14,0 16,0

Supplementary Figure 2| Fluorescence decay data and data analysis for live HeLa cells FLIM image. Upper panel shows the selection of 3 ROIs (5x5 pixels; A – cytoplasmic, B –nuclear, C –mitochondria). Below are the screenshots from Becker & Hickl SPCImage data acquisition and analysis window for each of the ROI shown. Blue dots of the main plot represent experimental decay data, red line shows exponential fit of the data, green curve denotes instrument response function (IRF). An additional plot in a bottom of each screenshot shows fitting residuals. In ROI A single component exponent (τ~2.2 ns) fits experimental data well, indicating presence of the single EGFP-actin signal. In ROIs B and C two-component model provides more adequate fitting unmixing EGFP-actin (τ_2 ~2.2 ns) from the shorter lifetime signals of T65G (τ_1~0.85 ns, nucleus) and BrUSLEE (τ_1~0.6 ns, mitochondria).

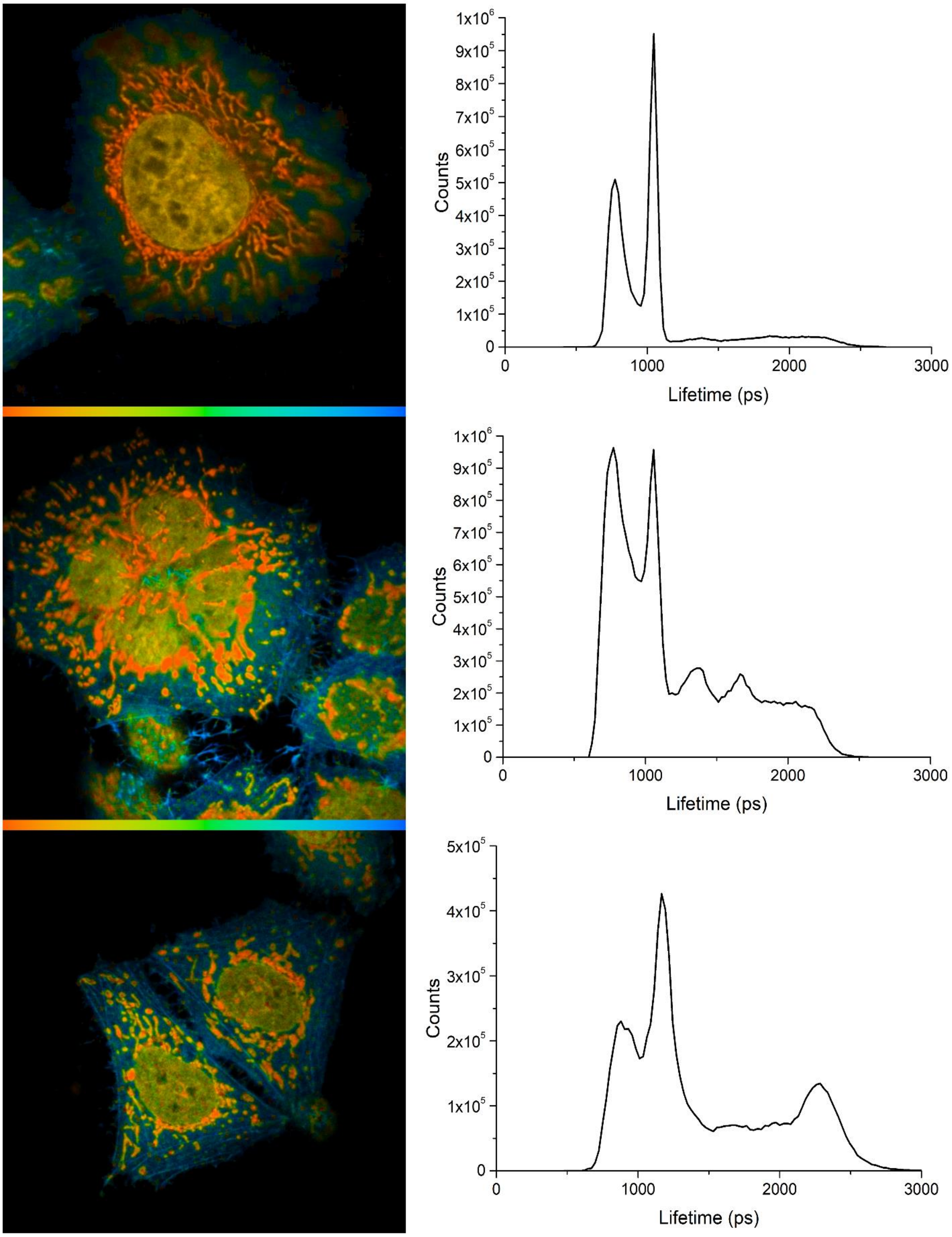

Supplementary Figure 3 | Color-coded FLIM images of live HeLa cells expressing EGFP-actin (mostly in the cytoplasm; τ~2.2 ns, Tm~2.2 ns), EGFP T65G-histone 2B (inside the nucleus; τ_1~0.85 ns, τ_2 ~2.2 ns, Tm ~1.1 ns), EGFP T65G/Y145M/F165Y-mito (inside the mitochondria; τ_1~0.6 ns, τ_2 ~2.2 ns, Tm=0.8 ns). Color-coded combined intensity+lifetime images are shown on the left. Color legend is shown as a horizontal bar between images and covers 750-2400 ps range. Corresponding lifetime distribution histograms are shown on the right. Horizontal axis sets mean lifetime (Tm, average lifetime in pixel), vertical axis sets photon counts. Single-photon fluorescence excitation at 488 nm was used to acquire these images.

Supplementary methods

Photostability evaluation. AF6000 LX microscope (Leica Microsystems, Wetzlar, Germany) with a 63× 1.4NA oil objective, a Photometrics (Tucson, AZ) CoolSNAP HQ CCD camera, and a 120W HXP short arc lamp (Osram, Munich, Germany) were used to acquire data. Purified proteins (EGFP, T65G, T65G-Y145M, BrUSLEE) were immobilized onto metal-affinity resin spherical particles (TALON ®, Clontech) prior to microscopy and imaged in PBS solution pH 7.4 (Gibco). For *in cellulo* experiments, human embryonic kidney 293 (HEK293T) cells were transfected with vectors encoding fluorescent proteins (EGFP, T65G, T65G-Y145M, BrUSLEE). Live cells were imaged 24-48 h post transfection at room temperature.

A field containing either several resin particles or cells was irradiated through GFP filter cube (excitation BP470/40, emission BP525/50) in a series of light pulses for detecting (25–50 mW/cm^2, exposure 10–100 ms) and bleaching (1.5 W/cm^2, exposure 5 s). The bleaching curves for individual particles/cells were normalized to the initial signal values. A time to 50% fluorescence drop (bleaching half-time) was used as a quantitative characteristic for photostability. Data were normalized within each experiment. For each protein, at least 3 independent experiments (3-5 particles or 10-15 cells in each experiment) were performed.
